# Combination of oral nonabsorbable and intravenous antibiotics versus intravenous antibiotics alone in the prevention of surgical site infections after elective colorectal surgery in pediatric patients: A retrospective study

**DOI:** 10.1097/MD.0000000000012288

**Published:** 2018-09-07

**Authors:** Xie Xiaolong, Wu Yang, Zheng Xiaofeng, Wang Qi, Xiang Bo

**Affiliations:** Department of Pediatric Surgery, West China Hospital, Sichuan University, Sichuan, China.

**Keywords:** elective colorectal surgery, oral nonabsorbable antibiotics, pediatric, surgical site infections

## Abstract

We conducted this study to compare the effectiveness of combined oral nonabsorbable and intravenous antibiotics versus intravenous antibiotics alone in reducing the incidence of surgical site infections following elective colorectal surgery in pediatric patients.

Between January 2010 and December 2016, patients from 0 to 14 who underwent elective colorectal surgery were retrospectively analyzed. Based on intravenous antibiotics with and without oral antibiotics, the patients were grouped as OA group (combination of oral nonabsorbable and intravenous antibiotics) or A group (the intravenous antibiotics alone). Neomycin combined with erythromycin was used in OA group. The data collected included demographic data, diagnosis, procedure being performed, operative time, time to first stool, time to removal of the nasogastric tube, time to full enteral feeds, hospital length of stay, and prophylactic antibiotics (days ± standard deviation). The main outcome was the rate of postoperative infectious complications, such as wound infection, anastomotic leak, and intra-abdominal abscess formation.

A total of 564 children who underwent elective colorectal surgery were enrolled which consist of OA group (combination of oral nonabsorbable and intravenous antibiotics) and A group (the intravenous antibiotics alone), the number of the former one was 216 and the latter one was 348. Postoperative complications were similar in both groups of patients. In the OA group, we observed 5 anastomotic leak, 6 wound infections, and 5 intra-abdominal abscesses. In the A group, we observed 13 anastomotic leak, 9 wound infections, and 11 intra-abdominal abscesses. Analysis with Fisher exact test revealed no statistically significant difference in the incidence of wound infection, anastomotic leak, and intra-abdominal abscess between the 2 groups.

The results of our study suggest that omitting oral nonabsorbable antibiotics before elective colorectal surgery in infants and children carries no increased risk of infectious or anastomotic complications.

## 1 Introduction

Surgical site infections (SSIs) are a costly and potentially preventable source of morbidity, representing the most common cause of hospital-acquired infection in the surgical population.^[1,2]^ Colorectal operations have been associated with the highest risk of SSI and other infectious complications owing to the heavy bacterial load of the colon and rectum.^[3,4]^

Since the introduction of sulfanilamide into clinical practice over 8 decades ago and the recognition that mechanical bowel preparation (MBP) did not reduce the concentration of colonic bacteria or SSI occurrence, surgeons have been exploring the utility of enteric administration of antibiotics for colonic decontamination.^[5,6]^ The advantage of adding oral nonabsorbable antibiotics to intravenous antibiotics to decrease SSI after colorectal surgery is not well known. The clinical benefit of enteral antibiotics combined with intravenous antibiotics has been further confirmed through several randomized trials and meta-analyses in adults. In what is currently the largest such meta-analysis, Bellows et al^[7]^ analyzed 16 trials including a total of 2669 patients.^[8]^ The authors found that the addition of enteral nonabsorbable antibiotics to standard parenteral antibiotic prophylaxis at the time of surgery reduced SSI risk by 43% compared with parenteral antibiotics alone.

Current recommendations on the utility of oral nonabsorbable antibiotics in pediatric surgery are based largely on adult literature. Data for the use of oral nonabsorbable antibiotics before elective colorectal surgery in pediatric patients are limited and the necessity of oral nonabsorbable antibiotics in this population remains unclear. Therefore we conducted this study to compare the effectiveness of combined oral nonabsorbable and intravenous antibiotics versus intravenous antibiotics alone in reducing the incidence of SSI following elective colorectal surgery in pediatric patients.

## 2 Methods

This retrospective cohort study was approved by the ethics committees of West China Hospital of Sichuan University (No. 417, November 18, 2017). Because of the retrospective nature of this study, our committee waived the need for patient consent. Candidates for inclusion in the study were children from 0 to 14 years who underwent elective colorectal surgery during the period from January 2010 to December 2016. Based on intravenous antibiotics with and without oral antibiotics, the patients were grouped as OA group (combination of oral nonabsorbable and intravenous antibiotics) or A group (the intravenous antibiotics alone), in which neomycin combined with erythromycin was used in OA group. Regimen of 1 g of neomycin and 1 g of erythromycin were given 3 times after bowel preparation the day before surgery.

Patients in both group received MBP with 25 mL/kg/h of polyethylene glycol 12 to 16 hours before surgery. All patients were allowed to have a regular diet until midnight the evening before surgery (patients usually took their mechanical preparation after the last solid meal). Both groups received 1 preoperative dose of intravenous cefoxitin 30 mg/kg, up to 2 g administered 30 minutes before skin incision, and 1 postoperative dose administered 8 hours from the first dose. For patients with penicillin or cephalosporin allergies, gentamicin 2.5 mg/kg and clindamycin 10 mg/kg were administered at equivalent time points. Surgeons were allowed to continue the prophylactic intravenous antibiotics for more than 1 day if necessary. Four surgeons were enrolled in the study, all with high specialization in colorectal surgery (>40 procedures/year).

The data collected included demographic data, diagnosis, procedure being performed, operative time, time to first stool, time to removal of the nasogastric tube (NGT), time to full enteral feeds, hospital length of stay (LOS), pophylactic antibiotics (days ± standard deviation), and 90-day postoperative follow-up were prospectively entered in a Microsoft Excel database. The main outcome was the rate of postoperative infectious complications, such as wound infection, anastomotic leak, and intra-abdominal abscess. Wound infection was defined as a wound requiring partial or complete opening for drainage of purulent collection, or erythema requiring initiation of antibiotic treatment. Anastomotic leak was identified if demonstrated by imaging or documented in surgery, or if fecal drainage was evident through a perianastomotic drain. Abdominal abscess was defined as fluid collection demonstrated by computed tomography scan, in conjunction with elevated temperature or white blood cell count.

### 2.1 Statistical analyses

Data collection was performed on a standardized, computerized, secured case-record form accessible online and was controlled by an independent data-management center. All statistical analysis was performed with the use of SPSS Statistics for Windows, version 23.0 (SPSS). The categorical descriptive data were reported as counts (N) and percentage (%). The categorical univariate analysis was done by Fisher exact test. The numerical descriptive data were reported as mean and standard deviation. The data were analyzed using the *χ*^2^ and the Student *t* test. The statistical significance level was set as 2 tailed with *P* value <.05.

## 3 Results

**Figure 1:**
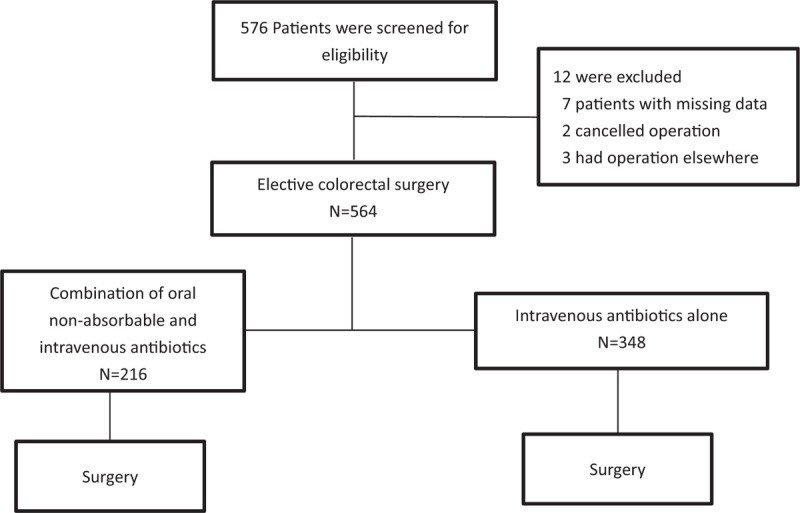
Algorithm of study protocol.

A total of 576 surgical interventions were identified among patients who underwent elective colorectal surgery in West China Hospital of Sichuan University. According to the retrospective study, missing data elements were identified in 7 records which were excluded. Two patients cancelled operation and 3 patients had operation elsewhere. Five hundred sixty-four surgical interventions were included in this study (Fig. 1).

The baseline characteristics of all the patients are shown in Table 1. Median follow-up time was 3 (2–4) months. The male to female ratio was 2.28:1. The median age of the patient was 27.00 (8.00–45.00) months with a mean weight of 13.38 kg. The patients in each group were similar in the distribution of age, sex, diagnoses, and type of procedure. Underlying diseases included Hirschsprung disease, imperforate anus, ulcerative colitis, Crohn disease, reestablishment of intestinal continuity with history of necrotizing enterocolitis, duplication cyst, meconium ileus, and colon atresia. Colostomy closure was the most common procedure in the OA group at 56.94% vs 60.63% of the A group. Proctectomy with pull-through for Hirschsprung disease comprised 12.04% of the OA group and 14.08% of the A group. The percentage of posterior sagittal anorectoplasty was similar in both groups (22.69% OA vs 17.24% A). The total operative time (75.67 ± 24.53 OA vs 76.23 ± 26.28 A, *P* = .800), the time to first stool (36.35 ± 15.15 OA vs 35.81 ± 14.69 A, *P* = .674), the time to removal of the NGT (44.19 ± 23.54 OA vs 45.82 ± 23.46 A, *P* = .423), time to full enteral feeds (46.71 ± 12.07 OA vs 47.37 ± 12.97 A, *P* = .607), hospital LOS (6.30 ± 1.62 OA vs 6.53 ± 2.11 A, *P* = .175), prophylactic antibiotics (1.91 ± 1.33 OA vs 1.91 ± 1.36 A, *P* = .110) in the OA group was not significantly different compared with A group.

**Table 1:**
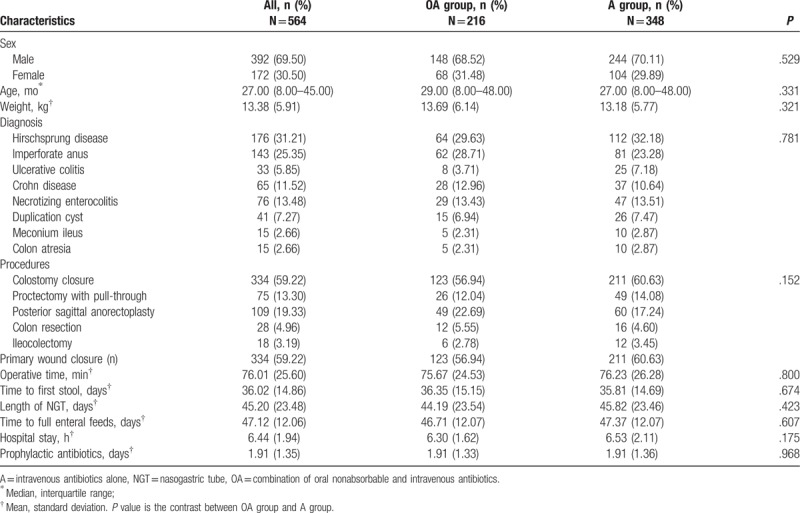
Baseline characteristics of the study population.

Complications categorized according to the Clavien-Dindo classification of surgical complications are presented in Table 2 and postoperative complications were similar in both groups of patients (Table 3). Analysis with Fisher exact test revealed no statistically significant difference in the incidence of wound infection, anastomotic leak, intra-abdominal abscess, and total number of complications between the 2 groups. In the OA group, we observed 5 anastomotic leak, 6 wound infections, and 5 abscesses. In the A group, we observed 13 anastomotic leak, 9 wound infections, and 11 abscesses. The leak in both groups occurred after colostomy closure and the 18 patients received re-exploration with revision of the anastomosis. All complications were managed with operative drainage, open wound care, antibiotics, and total parenteral nutrition. All patients with complications were eventually discharged and made uneventful recoveries after the management.

**Table 2:**
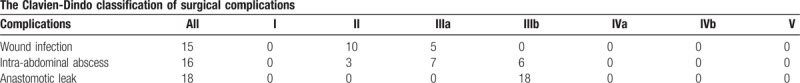
The Clavien-Dindo classification of surgical complications.

**Table 3:**
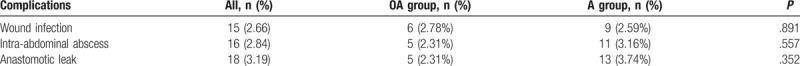
Complications of the study population.

## 4 Discussion

The aim of this study was to compare the effectiveness of combined oral nonabsorbable and intravenous antibiotics versus intravenous antibiotics alone in reducing the incidence of SSI following elective colorectal surgery in pediatric patients. Popularized by Nichols and Condon, the “standard” oral antibiotic regimen in colorectal surgery utilizes nonabsorbable antibiotics given the day before surgery.^[8]^ Although the use of prophylactic intravenous antibiotics in colorectal surgery in adults is considered the standard of care, the use of prophylactic oral antibiotics is more controversial.

A prospective, randomized clinical trial within the Veterans’ Administration showed that the use of oral nonabsorbable antibiotics (e.g., neomycin/erythromycin) significantly reduced the incidence of SSIs (9% vs 35%) and anastomotic leaks (0% vs 10%) compared with a placebo.^[9]^ More contemporary evidence to support the efficacy of combining oral nonabsorbable antibiotics with intravenous antibiotics has been shown in 2 large, multicenter prospective colorectal database studies.^[10,11]^ As part of the Michigan Surgical Quality Colectomy Best Practices Collaborative Study, Englesbe et al^[11]^ examined 2011 patients undergoing elective colorectal procedures at 24 hospitals between 2007 and 2009. Using propensity-matched analysis to adjust for factors that may have influenced the decision to use oral nonabsorbable antibiotics, as well as adjusting for patient, procedure, and process measure risk factors associated with SSI, the authors found a significant reduction in the rates of intra-abdominal abscesses (1.8% vs 4.2%, *P* = .044) and incisional SSIs (2.6% vs 7.6%, *P* = .001) when oral nonabsorbable antibiotics were combined with intravenous antibiotics compared to intravenous antibiotics alone. The rationale of both strategies being used together is that oral nonabsorbed antibiotics reduce the inoculum of bacteria contaminating the surgical site from the colon, and systemic antibiotics provide a safety net of effective drug in the soft tissues to minimize the risk of infection. Interestingly, the use of oral nonabsorbable antibiotics among colorectal surgeons has steadily declined from 86% in 1997 to 36% in 2010.^[12,13]^

Current recommendations on the utility of oral nonabsorbable antibiotics in pediatric surgery are based largely on adult literature and data for the use of oral nonabsorbable antibiotics before elective colorectal surgery in pediatric patients are limited. Studies investigated the use of oral nonabsorbable combined with systemic antibiotics showed conflicting results indicating that a study should be performed to determine whether oral nonabsorbable antibiotics, in combination with systemic antibiotics, are associated with a reduced incidence of SSI and anastomotic leaks compared with systemic antibiotics alone in pediatric patients. In our study, we found no significant differences between the 2 groups. We also found no differences in time to first stool, time to removal of the NGT, time to full enteral feeds, and hospital LOS.

Oral nonabsorbable antibiotics should be administered after a full MBP because the massive colonic burden of the intraluminal bacteria has to be greatly reduced for any effective local antimicrobial action to occur. In our center, our practice includes MBP, oral nonabsorbable antibiotics and perioperative intravenous antibiotics. Our study found that omitting oral nonabsorbable antibiotics before elective colorectal surgery in infants and children carries no increased risk of infectious or anastomotic complications. There are now several randomized controlled trials that suggest that MBP can be safely omitted in the majority of colorectal resections without increasing the incidence of SSIs.^[14]^

Eliminating oral nonabsorbable antibiotics may reduce the cost of healthcare and inconvenience for these patients, without compromising outcomes. These findings warrant a large, prospective, randomized clinical trial to validate our findings and to investigate further the necessity of oral nonabsorbable antibiotics in the pediatric population.

## 5 Conclusion

The results of our study suggest that omitting oral nonabsorbable antibiotics before elective colorectal surgery in infants and children carries no increased risk of infectious or anastomotic complications.

## Author contributions

**Conceptualization:** Xiang Bo.

**Data curation:** Xie Xiaolong, Wu Yang, Wang Qi.

**Formal analysis:** Wu Yang, Xiang Bo.

**Investigation:** Xie Xiaolong, Wu Yang.

**Methodology:** Wu Yang, Zheng Xiaofeng.

**Project administration:** Xie Xiaolong, Wu Yang.

**Resources:** Zheng Xiaofeng.

**Software:** Xie Xiaolong, Wu Yang, Wang Qi.

**Supervision:** Zheng Xiaofeng.

**Validation:** Wang Qi.

**Visualization:** Xie Xiaolong, Xiang Bo.

**Writing – original draft:** Xie Xiaolong.

**Writing – review and editing:** Xiang Bo.
